# Construction and Validation of a Risk Prediction Model for Acute Gastrointestinal Injury in Non-ICU Elderly Critically Ill Patients

**DOI:** 10.1007/s11606-025-09573-9

**Published:** 2025-05-08

**Authors:** Jiajia Xu, Shan Li, Yue Hu, Dan Liu, Jianghong Zhang, Binrong Zhang, Sisi Yuan, Xiaohong Zhang

**Affiliations:** 1 General Medical Department, The Third Hospital of Shanxi Medical University (Shanxi Bethune Hospital), Taiyuan City, China; 2 College of Nursing, Shanxi Medical University, Taiyuan City, China; 3 Nursing Department, The Third Hospital of Shanxi Medical University (Shanxi Bethune Hospital), Taiyuan City, China

**Keywords:** elderly, acute gastrointestinal injury, non-ICU, critical illness, risk prediction model, nomogram

## Abstract

**Background:**

Acute gastrointestinal injury (AGI) has a relatively high prevalence among elderly critically ill patients in non-intensive care units (non-ICUs), and significantly influences their clinical outcomes. Therefore, it is important to identify people at risk for AGI and take preventive measures as early as possible.

**Objective:**

We aimed to construct and validate a risk prediction model for AGI in non-ICU elderly critically ill patients.

**Design:**

Case–control study.

**Participants:**

In total, 538 elderly critically ill patients admitted to the general medical department of a tertiary hospital in Shanxi from April 2021 to May 2024.

**Main Measures:**

Influential factors for AGI were determined using univariate and multifactorial logistic regression analyses. We constructed a risk prediction model and created a nomogram. The bootstrap resampling method was utilized for internal validation. A total of 151 patients from different time periods were selected for the external validation.

**Key Results:**

The multifactorial logistic regression analysis revealed that the independent predictors for AGI were the duration of antibiotic use, number of vasoactive drugs, delayed enteral nutrition, age-corrected Charlson comorbidity index, and white blood cell count, all of which were included in the model and created a nomogram. The Omnibus test showed that the overall efficacy of the model was good (*P* < 0.001). The area under the receiver operating characteristic curve (AUC) was 0.807, the corrected AUC was 0.806, and the AUC was 0.796 for external validation, indicating good model discrimination. The calibration curves and Hosmer–Lemeshow tests revealed that the model was well calibrated (*P* = 0.627, Brier = 0.172 in internal validation; and *P* = 0.366, Brier = 0.182 in external validation). The clinical decision curves showed that the model had good clinical utility.

**Conclusions:**

AGI is common in non-ICU elderly critically ill patients. This AGI risk prediction model can be used as a screening tool to identify high-risk patients for AGI and assist clinical decision making.

**Supplementary Information:**

The online version contains supplementary material available at 10.1007/s11606-025-09573-9.

## INTRODUCTION

With the acceleration of global population ageing, the number of elderly critically ill patients is increasing and their health management faces many challenges^1^. Studies have shown that acute gastrointestinal injury (AGI) is present in 20–85% of elderly critically ill patients^2^. If not recognized and managed promptly, AGI may affect nutritional status and even trigger systemic inflammatory response and multi-organ dysfunction, which seriously threaten patients’ health^3^.

In Chinese clinical practice, elderly critically ill patients are distributed among two different healthcare settings, including ICU wards and non-ICU wards. Non-ICU elderly critically ill patients are those aged 60 years and above, who have severe conditions with a high risk of deterioration but do not require transfer to the ICU due to relatively stable vital signs. They need to undergo close monitoring and active treatment in general wards^4,5^. The cross-sectional pre-survey we conducted prior to this study found that the incidence of AGI in non-ICU elderly critically ill patients was as high as 40%, further highlighting the prevalence of AGI in this population.

However, there are relatively few studies on AGI in non-ICU elderly critically ill patients, lacking both systematic research on its influencing factors and few comprehensive quantitative evaluations^6–8^. In addition, compared with ICU patients, there are significant differences in the clinical status of non-ICU elderly critically ill patients in terms of pathophysiologic characteristics, intervention strategies, and monitoring frequency^5,9^. This has led to the fact that previous AGI models constructed for ICU geriatric critically ill patients are not applicable to non-ICU geriatric critically ill patients, manifesting heterogeneity in key dimensions such as population characteristics, potential risk factors, and study objectives. Therefore, our study focused on non-ICU elderly critically ill patients, aiming to explore the influencing factors of AGI and to construct a risk prediction model suitable for clinical application, in order to realize early identification and precise intervention for the high-risk group of AGI and to improve the prognosis of patients.

## METHODS

### Participants

A total of 538 elderly critically ill patients admitted to the general medical department of a tertiary hospital in Shanxi from April 2021 to May 2024 were selected as the study population (Fig. S1). Among them, 387 patients admitted from April 2021 to May 2023 served as the derivation cohort, and 151 patients admitted from June 2023 to May 2024 served as the validation cohort. The inclusion criteria were as follows: (1) aged 60 and above; (2) length of hospitalization ≥ 48 h; and (3) notification of serious illness in the doctor’s orders. The exclusion criteria were as follows: (1) clinical information was severely lacking (missing more than 20%)^10^; (2) severe gastrointestinal disease or history of gastrointestinal surgery at the time of admission to the hospital; (3) the presence of significant AGI on admission to the hospital; (4) non-resuscitative end-of-life state; and (5) patients who were in ICUs or who had met the admission criteria of the ICU but had not been transferred to the ICU due to resource constraints or personal wishes, etc.

Following the 10EVP sample size estimation method of logistic regression, the sample size should be at least 10 times the number of independent variables^11^. In this study, 30 independent variables were expected to be included, and the incidence of AGI in elderly critically ill patients ranged from 20 to 85%^2^. Thus, the required sample size was at least 353 cases. The derivation cohort included 387 patients, which met the requirements. This study was reviewed and approved by the Ethics Committee of the Shanxi Bethune Hospital (YXLL-2023–273).

### Baseline Predictor Variables and Outcome Measures

We summarized the results of relevant literature on AGI in critically ill elderly patients by reviewing the literature and combining them with the clinical situation to develop a data collection form. These variables included age, sex, smoking, drinking, primary disease diagnosis, high blood pressure, diabetes, respiratory failure, kidney insufficiency, Barthel index, modified early warning score (MEWS), age-corrected Charlson comorbidity index (ACCI), delayed enteral nutrition (EN), number of vasoactive drugs, number of antibiotics, the duration of antibiotic use, glucocorticoids, nonsteroidal anti-inflammatory medicines, sedative medications, mechanical ventilation, oxygen partial pressure, serum sodium, blood lactate, aspartate aminotransferase, albumin, blood creatinine, D-dimer, white blood cell (WBC), hemoglobin, and C-reactive protein (CRP). Concerning the Chinese Guidelines for the Clinical Application of Parenteral Enteral Nutrition in Adults^12^ and preliminary investigation, delayed EN was defined as insufficient intake (less than 60% of the target requirement for more than 7 days cumulatively before and after admission), without appropriate nutritional supportive therapy. The ACCI score was calculated as shown in Table S1. The dichotomous variable “whether AGI occurred” was used as the outcome variable (assigned values: 1 = AGI and 0 = no AGI). Referring to the grading of gastrointestinal injury symptoms proposed by the European Society of Intensive Care Medicine in 2012 regarding AGI^13^, grades I–IV were considered as if the patient had AGI (positive outcome) (Table S2).

### Statistical Analysis

Epidata software and SPSS 25.0 software were used to enter and analyze the data. The multiple interpolation method was used to fill in the missing data. Continuous variables were described as median (interquartile ranges (IQR)) and compared using the Mann–Whitney *U* test. Categorical variables were presented as proportions and were compared using chi-square or Fisher’s exact tests. Variables that were statistically significant in the univariate analysis were subjected to multicollinearity diagnosis. Variables without multicollinearity were taken as candidates to enter the logistic regression analysis (forward: LR) to determine the final predictor variables. A nomogram risk prediction model was constructed using the R software. The Omnibus test was used to evaluate the overall efficacy of the model. The bootstrap 1000 resampling method was employed for internal validation. In total, 151 non-ICU elderly critically ill patients newly included were used for external validation. Receiver operating characteristic (ROC) curves, calibration curves combined with the Hosmer–Lemeshow test and Brier index, and clinical decision curves were used to evaluate the model’s discrimination, calibration, and clinical utility. All statistical tests were two-sided and *P* < 0.05 was regarded as statistically significant (Fig. S2).

## RESULTS

### Patient Characteristics in Derivation and Validation Cohorts

The derivation cohort included 387 non-ICU elderly critically ill patients, of whom 235 (60.72%) did not have AGI and 152 (39.28%) did (71 in AGI grade I, 79 in AGI grade II, 2 in AGI grade III, and 0 in AGI grade IV). The validation cohort included 151 non-ICU elderly critically ill patients, of whom 88 (58.28%) did not experience AGI and 63 (41.72%) did (32 in AGI grade I, 30 in AGI grade II, 1 in AGI grade III, and 0 in AGI grade IV). Baseline comparisons of clinical information between the patients in the derivation and validation cohorts were not statistically significant and were balanced and comparable (*P* > 0.05) (Table S3).

### Univariate Analysis

The results of the univariate analysis showed that age, diabetes, Barthel index, MEWS, ACCI, delayed EN, number of vasoactive drugs, number of antibiotics, the duration of antibiotic use, sedative medication, serum sodium, aspartate aminotransferase, albumin, D-dimer, WBC, hemoglobin, and CRP were correlated with AGI (*P* < 0.05) (Table 1).

**Table 1:** The Univariate Analyses of the Predictive Factors Associated with AGI

Variable	Category	Non-AGI group (*n* = 235)	AGI group (*n* = 152)	*P* value
Age (years), median (IQR)		83 (72,87)	85 (76,89)	0.030*
Sex, *n* (%)				0.513
	Female	77 (32.8)	45 (29.6)	
Smoking, *n* (%)				0.076
	Current	28 (11.9)	28 (18.4)	
Drinking, *n* (%)				0.230
	Current	34 (14.5)	29 (19.1)	
Primary disease diagnosis,* n* (%)				0.229
	Respiratory system	125 (53.2)	91 (59.9)	
	Circulatory system	27 (11.5)	14 (9.2)	
	Nervous system	30 (12.8)	17 (11.2)	
	Endocrine system	1 (0.4)	1 (0.7)	
	Urinary system	7 (3.0)	3 (2.0)	
	Systemic reactions	14 (6.0)	16 (10.5)	
	Other	31 (13.2)	10 (6.6)	
High blood pressure, *n* (%)				0.904
	Yes	147 (62.6)	96 (63.2)	
Diabetes, *n* (%)				0.001*
	Yes	56 (23.8)	61 (40.1)	
Respiratory failure, *n* (%)				0.105
	Yes	71 (30.2)	58 (38.2)	
Kidney insufficiency, *n* (%)				0.886
	Yes	45 (19.1)	30 (19.7)	
Barthel index, *n* (%)				0.001*
	Severe/complete dependence	160 (68.1)	126 (82.9)	
ACCI, median (IQR)		6 (5, 7)	7 (6, 8)	< 0.001*
MEWS, median (IQR)		2 (1, 2)	2 (1, 4)	0.001*
Oxygen partial pressure (mmHg), *n* (%)				0.831
	< 80	149 (63.4)	98 (64.5)	
Serum sodium (mmol/L), *n* (%)				0.013*
	135–145	139 (59.1)	77 (50.7)	
	< 135	92 (39.1)	64 (42.1)	
	> 145	4 (1.7)	11 (7.2)	
Blood lactate (mmol/L), *n* (%)				0.510
	> 1.7	111 (47.2)	77 (50.7)	
Aspartate aminotransferase (IU/L), *n* (%)				0.046*
	> 40	34 (14.5)	34 (22.4)	
Albumin (g/L), *n* (%)				0.014*
	< 35	138 (58.7)	108 (71.1)	
Blood creatinine (μmol/L), *n* (%)				0.139
	> 133	29 (12.3)	27 (17.8)	
D-dimer (ng/mL), *n* (%)				0.023*
	> 500	113 (48.1)	91 (59.9)	
White blood cells (× 10^9^/L), *n* (%)				0.004*
	> 10	47 (20.0)	50 (32.9)	
Hemoglobin (g/L), *n* (%)				0.031*
	< 120 (male)/< 110 (female)	140 (59.6)	107 (70.4)	
C-reactive protein (mg/L), *n* (%)				0.007*
	> 10	163 (69.4)	124 (81.6)	
Mechanical ventilation, *n* (%)				0.896
	Yes	39 (16.6)	26 (17.1)	
Delayed enteral nutrition, *n* (%)				< 0.001*
	Yes	89 (37.9)	98 (64.5)	
Number of vasoactive drugs (kinds), median (IQR)		0 (0, 0)	0 (0, 1)	< 0.001*
Number of antibiotics (kinds), median (IQR)		1 (0, 2)	2 (1, 2)	< 0.001*
Duration of antibiotic use (days), *n* (%)				< 0.001*
	> 7	80 (34.0)	102 (67.1)	
Glucocorticoid, *n* (%)				0.519
	Yes	67 (28.5)	48 (31.6)	
Nonsteroidal anti-inflammatory medicine, *n* (%)				0.257
	Yes	53 (22.6)	42 (27.6)	
Sedative medication, *n* (%)				0.012*
	Yes	71 (30.2)	65 (42.8)	

*ACCI* age-corrected Charlson comorbidity index, *MEWS* modified early warning score

*P-value < 0.05

### Multicollinearity Diagnosis

The diagnosis of multicollinearity explores whether there is a high degree of correlation between variables, using the variance inflation factor (VIF) or tolerance (TOL) to determine whether multicollinearity exists between variables. In this study, multicollinearity diagnostic tests were performed on variables with statistical significance in the univariate analysis, as detailed in Table S4. The results showed that multicollinearity was not evident among the 17 statistically significant independent variables in the univariate analysis (VIF < 5 and TOL > 0.2).

### Multifactorial Logistic Regression Analysis

The results of the logistic regression analysis showed that five variables entered the model, which were the duration of antibiotic use (OR 3.197, 95%CI 1.961–5.212, *P* < 0.001), number of vasoactive drugs (OR 1.465, 95%CI 1.056–2.032, *P* = 0.022), delayed EN (OR 3.452, 95%CI 2.096–5.687, *P* < 0.001), ACCI (OR 1.455, 95%CI 1.276–1.659, *P* < 0.001), and WBC (OR 2.145, 95%CI 1.243–3.701, *P* = 0.006) (Table 2). Among them, the variables “delayed EN” (no or yes), “duration of antibiotic use” (≤ 7 days or > 7 days), and “WBC” (≤ 10 × 10^9^/L or > 10 × 10^9^/L) were dichotomous variables; the variables “ACCI” and “number of vasoactive drugs” were continuous variables.

**Table 2:** The Multifactorial Logistic Regression Analysis of the Potential Predictive Factors Associated with AGI

Variable	*β*	SE	*P* value	OR (95% CI)
ACCI	0.375	0.067	< 0.001	1.455 (1.276–1.659)
Duration of antibiotic use	1.162	0.249	< 0.001	3.197 (1.961–5.212)
Number of vasoactive drugs	0.382	0.167	0.022	1.465 (1.056–2.032)
Delayed enteral nutrition	1.239	0.255	< 0.001	3.452 (2.096–5.687)
WBC	0.763	0.278	0.006	2.145 (1.243–3.701)
Constant	− 4.450	0.544	< 0.001	

*β* biased regression coefficient, *SE* standard error tolerance, *OR* odds ratio, *CI* confidence interval, *ACCI* age-corrected Charlson comorbidity index, *WBC* white blood cell

### Model Construction

**Figure 1:**
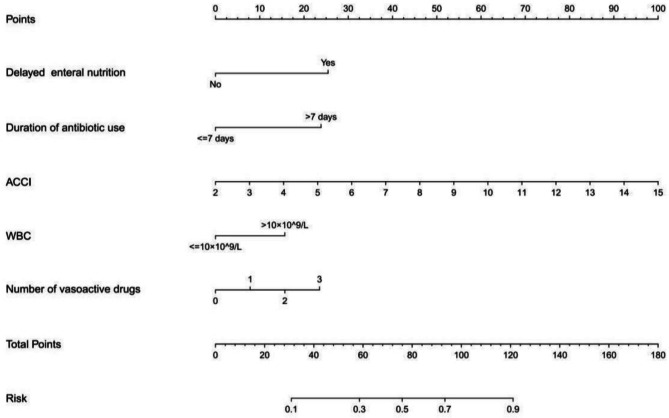
Nomogram predicting the probability of AGI in non-ICU elderly critically ill patients.

We constructed a predictive model for the risk of AGI in non-ICU elderly critically ill patients based on the biased regression coefficients of the independent variables in the logistic regression equation, which was calculated as logit (*P*) = − 4.450 + 0.375 × ACCI + 1.239 × delayed enteral nutrition + 0.382 × number of vasoactive drugs + 1.162 × the duration of antibiotic use + 0.763 × WBC. The predictive model was presented as a nomogram that was used to quantitatively predict the risk probability of AGI among elderly critically ill patients in non-ICUs (Fig. 1). For example, an elderly critically ill patient in non-ICUs, whose admitted WBC count was 8 × 10^9^/L, the number of vasoactive drugs was 2, enteral nutrition was delayed, ACCI was 7, and the duration of antibiotic use was 10 days and had a risk probability of 79.2% of developing AGI, as shown in Fig. S3.

### Model Evaluation

**Figure 2:**
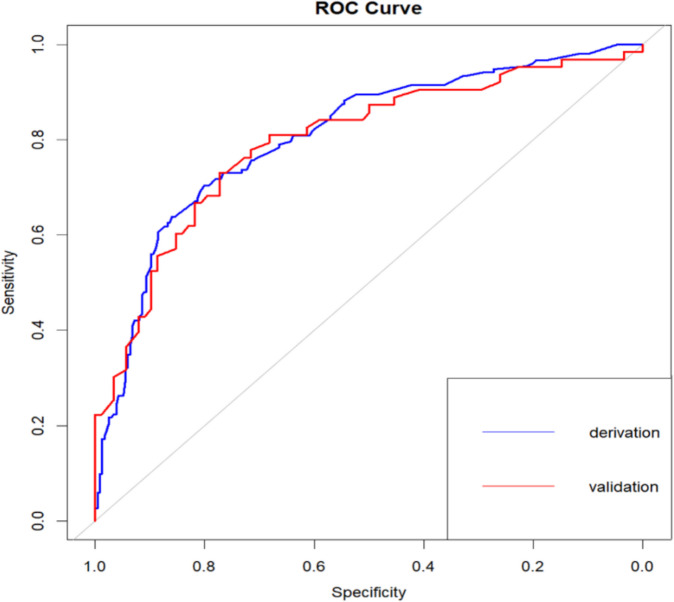
Receiver operating characteristic curve for the risk prediction model of AGI in the derivation and validation cohort.

**Figure 3:**
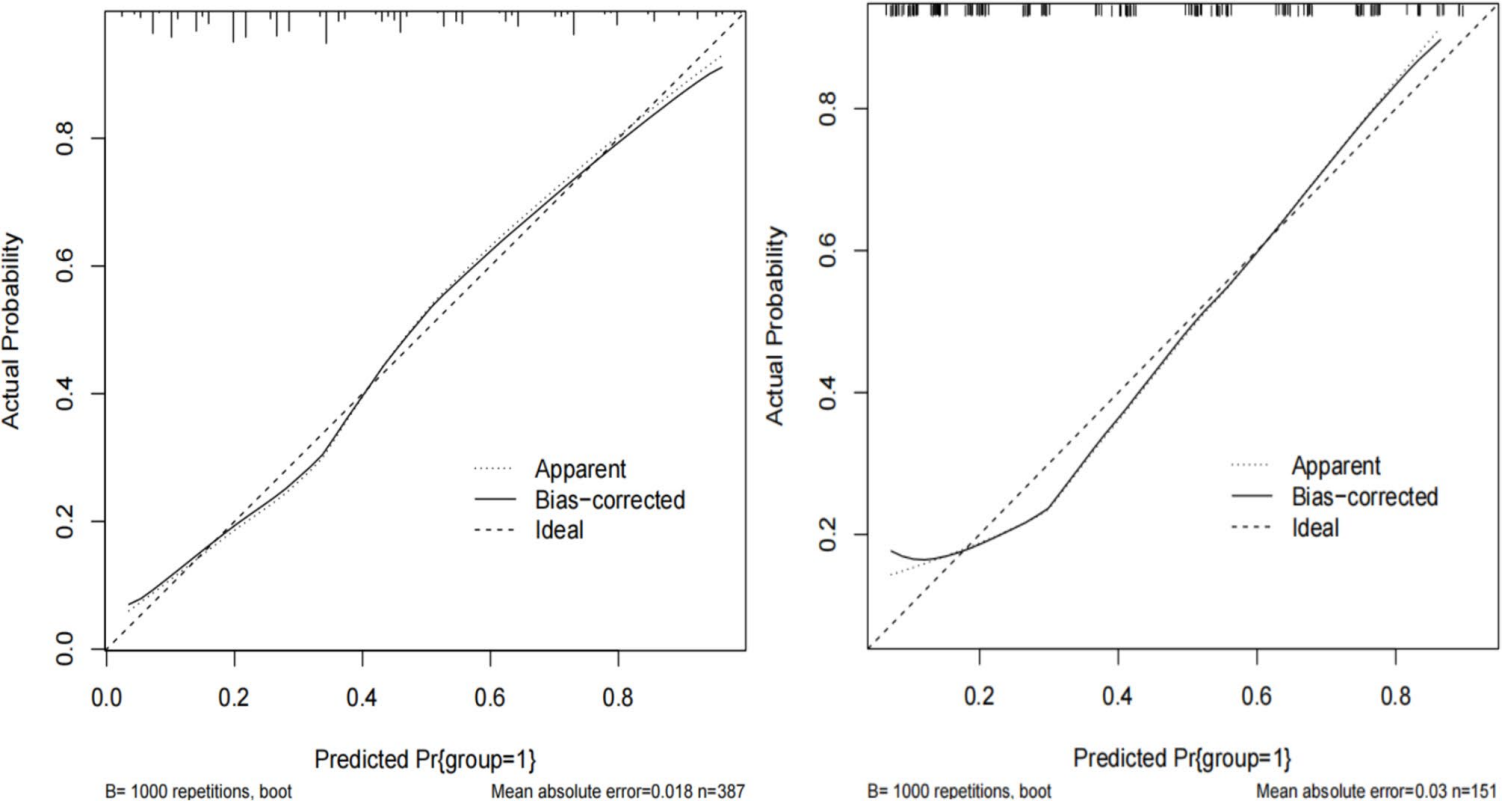
Calibration curve for the risk prediction model of AGI. The derivation cohort is on the left and the validation cohort is on the right.

**Figure 4:**
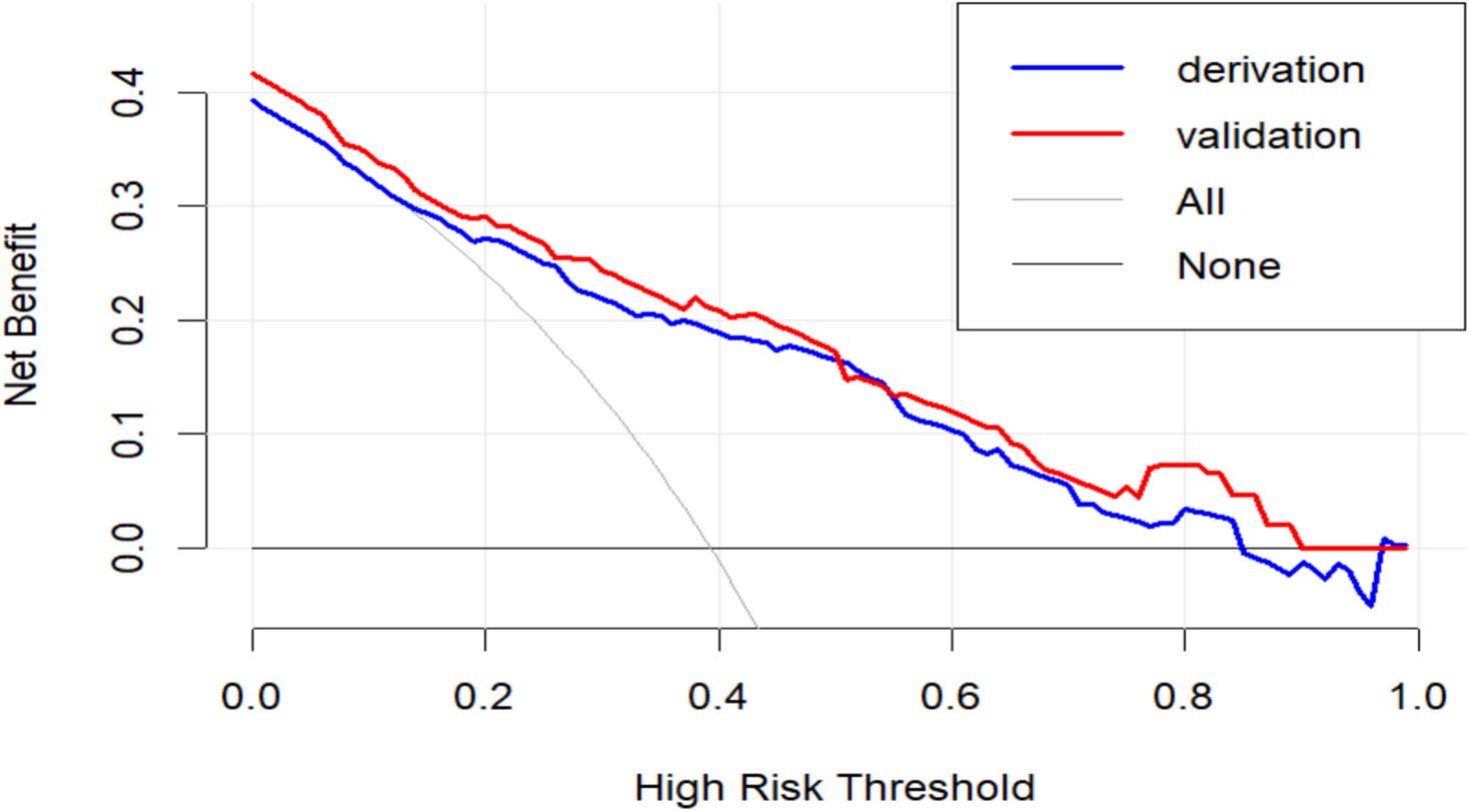
Clinical decision curve for the risk prediction model of AGI in the derivation and validation cohort.

The Omnibus test showed that the overall performance of the model was excellent (*P* < 0.001). The areas under the ROC curve (AUC) were 0.807 (95%CI: 0.762–0.852) in the derivation cohort with a sensitivity of 70.4% and a specificity of 80.0%, and 0.796 in the validation cohort, indicating that the model had a good degree of differentiation (Fig. 2). The 1000 ROC curves were concentrated around the average ROC curve, with a corrected AUC of 0.806, indicating a relatively stable model performance (Fig. S4). The calibration curve fitted the ideal curve well (Fig. 3). In addition, the *P*-value of the Hosmer–Lemeshow test was 0.627 and the Brier index was 0.172 for internal validation, and the *P*-value of the H–L test was 0.366 and the Brier index was 0.182 for external validation, indicating that the model was well calibrated. The clinical decision curve showed that the model had good clinical utility (Fig. 4).

## DISCUSSION

In this study, the incidence of AGI among non-ICU elderly critically ill patients was 39.28%, which was consistent with the previous report^2^.AGI is strongly associated with malnutrition, SIRS, MODS, and death^7^. Therefore, identifying individuals at high risk for AGI is essential to improve the prognosis of non-ICU elderly critically ill patients. The predictive efficacy of the model we constructed was excellent, with good discrimination, calibration, and clinical utility.

Consistent with the findings of a previous study^14^, we observed that the number of vasoactive drugs was significantly associated with AGI. Although vasoactive drugs are effective in improving cardiac coronary perfusion pressure, they exacerbate gastrointestinal ischemia and hypoxia, and indirectly stimulate the growth of Shiga toxin-producing *Escherichia coli*and other harmful flora, which severely damages the gastrointestinal mucosa and increases the risk of gastrointestinal dysfunction and failure^15^. In addition, patients with a duration of antibiotic use > 7 days were prone to AGI. It has been found that the use of antibiotics for more than 1 week can have an impact on the gut microbiome.^16^ Antibiotics destroy beneficial bacteria while killing pathogenic bacteria, inducing intestinal flora dysbiosis and even leading to systemic immune dysfunction^16,17^. Healthcare professionals should fully understand the mechanism of action of the above drugs and use them appropriately, as well as use microecological agents to maintain microecological balance when necessary. In addition, further development of novel drugs with less impact on gastrointestinal blood flow may be needed for elderly patients.

The results of this study showed that delayed EN was a risk factor for AGI. Clinical nutrition guidelines at home and abroad state that EN should be initiated as early as possible in critically ill patients in the absence of contraindications to enteral nutrition^18,19^. Compared with delayed EN, early EN provides clinical benefits by maintaining the gastrointestinal barrier and immune function, providing the body with metabolic substrates, and reducing the risk of death^20^. We found that elderly patients were unable to initiate appropriate EN therapy as early as possible because of feelings such as fear, shallow awareness of the association between their intake and health, and insufficient attention paid by healthcare professionals to nutritional support therapy in elderly patients. Therefore, they are often at risk of inadequate food intake. Bekebrede’s study showed that gastrointestinal energy deficiency due to inadequate food intake is an important cause of aggravated gastrointestinal dysfunction such as diarrhea^21^. Approximately 70% of the nutrient supply to the gastrointestinal mucosa comes from intestinal luminal nutrition, and chronic insufficient intake leads to a lack of adequate nutrient substrates in the gastrointestinal lumen, which affects the metabolism, repair, and regeneration of the mucosa. In addition, it increases the risk of malnutrition, and makes the gastrointestinal tract more vulnerable to pathogens, increasing the risk of AGI^22–24^. It is worth noting that for elderly critically ill patients with chronically insufficient intake, routine nasal feeding is highly likely to trigger enteral feeding intolerance and aggravate gastrointestinal burden^25^. Nutritional awareness should be raised among healthcare professionals and patients in clinical practice. Small-dose, low-rate enteral nutrition should be initiated as early as possible, and the amount and rate of feeding should be gradually increased to enhance gastrointestinal adaptability and meet the nutritional needs of patients^26^.

We found that WBC was a predictor of an elevated risk of AGI in elderly critically ill patients, consistent with the results of Sun’s study^27^. An increased WBC count implies an extremely active inflammatory response in the body^28^. The released inflammatory factors can activate the signal transduction of nuclear factors in gastrointestinal mucosal epithelial cells, which, together with bacterial invasion and immune imbalance, undermines the gastrointestinal barrier, resulting in a vicious cycle between systemic infection and AGI^29^. The association between ACCI and AGI is a novel finding. ACCI has been widely used to standardize the assessment of the degree of comorbidity and prognosis in elderly patients^30^. Respiratory, circulatory, and other systemic diseases induce gastrointestinal mucosal barrier damage, gastrointestinal motility disorders, and abnormal immune detection due to mechanisms such as insufficient gastrointestinal perfusion, changes in hormone levels, disruption of the internal environment of the bacterial flora, and abnormal autonomic regulation^31–33^. Systemic diseases such as sepsis and multiple injuries cause inflammatory responses due to infections, which are prone to hemodynamic instability and imbalance of the immune environment under strong stress, leading to edema and hemorrhage, as well as cellular detachment and necrosis of the gastrointestinal mucosa^3,34^. Age is also an important component of ACCI. Physiological aging leads to changes such as a decrease in the number of neurons in the gastrointestinal tract, prolonged peristalsis, and dysbiosis of the microflora^35^. It is recommended that healthcare professionals should aggressively treat underlying diseases to reduce the risk of AGI.

Among the five predictors screened, the association between delayed EN and AGI outcomes was stronger and more significant. From the perspective of actual clinical intervention benefits and intervention difficulty, delayed EN has more direct clinical intervention benefits and relatively lower intervention difficulty and risk compared with the other four predictive factors, and should be highly valued in clinical work. Consequently, from the perspective of EN, we can conduct subgroup analysis based on the key factor of timing of EN initiation and develop a standardized and systematic prevention program based on the results, which will play a vital role in effectively reducing the occurrence of AGI in elderly patients and improving the quality of life of elderly patients.

Moreover, we recommend using the model for risk prediction and dynamic monitoring of patients at the initial stage of the patients’ admission to the hospital and at critical junctures during the hospitalization, such as postoperative or changes in condition, to help physicians quickly understand the overall risk profile of patients and guide clinical decision-making. In addition, to ensure the applicability and accuracy of the model, it is recommended that external validation be performed in different medical centers or patient populations and that the model be adjusted and optimized as necessary based on the results.

Our study has a number of strengths. First, our model, constructed with the help of nomogram, improves the readability and accessibility of the model results. Second, our model not only enriches the research in the field of AGI risk prediction modeling, but also provides early warning and dynamic monitoring of AGI in the group of non-ICU elderly critically ill patients. This not only provides a reference for adopting or adjusting refined therapeutic measures to effectively prevent the progression of AGI and reduce the probability of ICU transfer, but also optimizes the allocation of healthcare resources, thereby improving the overall quality of care. Third, compared with the ICU model, the selection of variables in our model is closer to the actual resource conditions and routine clinical needs of non-ICU wards, thus reflecting real clinical practice and being more applicable to non-ICU elderly critically ill patients.

However, there are some limitations. Firstly, this was a single-center study, and the model was constructed based on data from patients at the specific time and location, so its generalizability needs to be further validated. Secondly, due to the limitations of sample size and data, this study was unable to finely classify different grades of AGI. In the future, we will carry out multi-center validation to further verify the generalization ability and clinical applicability of the model so that it can be adjusted and optimized accordingly in a timely manner. In addition, we will expand the sample size and develop a multi-classification prediction model to more accurately predict the risk probability of different grades of AGI, which will provide more powerful support for clinical decision-making.

## CONCLUSIONS

The incidence of AGI in non-ICU elderly critically ill patients was relatively high, and the predictive efficacy of the AGI risk prediction model was favorable. The model constructed in this study can be used as a screening tool for AGI in non-ICU elderly critically ill patients, effectively predicting the high-risk group and providing a basis for implementing individualized intervention strategies to improve the clinical outcomes of patients.

## Supplementary Information

Below is the link to the electronic supplementary material.

Supplementary file1 (DOCX 388 KB)

## Data Availability

The data in this study are available from the corresponding author on a reasonable request.

## Abbreviations

ICUs: Intensive care units
AGI: Acute gastrointestinal injury
ACCI: Age-corrected Charlson comorbidity index
MEWS: Modified early warning score
EN: Enteral nutrition
ROC: Receiver operating characteristic curve
WBC: White blood cell
CRP: C-reactive protein
VIF: Variance inflation factor
TOL: Tolerance
OR: Odds ratio
CI: Confidence interval
