# Supplementary material for: Construction and Validation of a Risk Prediction Model for Acute Gastrointestinal Injury in Non-ICU Elderly Critically Ill Patients
Source: J Gen Intern Med. 2025 May 8;40(11):2643–51. doi: 10.1007/s11606-025-09573-9 (PMC12405112; doi:10.1007/s11606-025-09573-9)
Supplement: Supplementary file 1 — Supplementary file1 (DOCX 388 KB) [file 11606_2025_9573_MOESM1_ESM.docx]

**SUPPLEMENTARY MATERIAL**

Construction and validation of a risk prediction model for acute gastrointestinal injury in non-ICU elderly critically ill patients

**Table S1. Age-Corrected Charlson Comorbidity Index Scoring Rubric.**

**Table S2. Definition and grading of acute gastrointestinal injuries.**

**Table S3. Baseline comparisons of clinically relevant information in cohorts.**

**Table S4. Multicollinearity diagnosis.**

**Figure S1. Flowchart of patient recruitment.**

**Figure S2. Flowchart of statistical methods.**

**Figure S3. Individual nomogram predicting the probability of AGI.**

**Figure S4. ROC for model of AGI in the derivation cohort (Bootstrap 1000 times resampling method).**

**Table S1 Age-Corrected Charlson Comorbidity Index Scoring Rubric**

| Score | Classification of Diseases and Age |
| --- | --- |
| 1 score | Myocardial infarction, Congestive heart failure, Peripheral vascular disease, Dementia, Cerebrovascular disease, Connective tissue disease, Mild liver disease, Peptic ulcer, Diabetes mellitus (without complications and end-organ damage), Age 50-59. |
| 2 scores | Hemiplegia, Moderate to severe chronic kidney disease, Diabetes mellitus (with complications and end-organ damage), Solid tumors (without metastasis), Leukemia, Lymphoma, Age 60-69. |
| 3 scores | Moderate to severe liver disease, Age 70-79. |
| 4 scores | Age ≥80. |
| 6 scores | Solid tumors (with metastasis), Acquired immunodeficiency syndrome. |

ACCI score was the sum of the comorbidity score and age score. Online tool for ACCI score: [https://www.mdcalc.com/charlson-comorbidity-index-cci](https://www.mdcalc.com/charlson-comorbidity-index-cci). Related Literature: Lin H, Xi YB, Yang ZC, et al. Optimizing Prediction of In-Hospital Mortality in Elderly Patients With Acute Myocardial Infarction: A Nomogram Approach Using the Age-Adjusted Charlson Comorbidity Index Score. J Am Heart Assoc. 2024;13(14):e032589.

**Table S2 Definition and grading of acute gastrointestinal injuries**

| **Grading** | **Definition** | **clinical manifestation** |
| --- | --- | --- |
| Non-AGI | Without gastrointestinal insufficiency | Without gastrointestinal symptoms |
| AGI grade I | Risk of progression to gastrointestinal dysfunction or failure: partial impairment of gastrointestinal function | Gastrointestinal symptoms of known cause or transient, transient and self-limiting, such as nausea, vomiting and abnormal bowel sounds on the first day after abdominal surgery. |
| AGI grade Ⅱ | Gastrointestinal dysfunction: inability of the digestive tract to adequately complete digestion and absorption to meet the body's needs for nutrients and water | Acute onset of gastrointestinal symptoms requiring clinical intervention. Examples include gastroparesis with gastric retention or reflux, low gastrointestinal paralysis, diarrhea, grade I intra-abdominal hypertension, visible bleeding from stomach contents or stools, and feeding intolerance. |
| AGI grade Ⅲ | Gastrointestinal failure: loss of gastrointestinal function, inability to regain gastrointestinal function after clinical intervention, no improvement in general condition | Clinical manifestations include feeding intolerance persists despite treatment - high gastric retention, persistent gastrointestinal paralysis, onset or progression of intestinal dilatation, progression of intra-abdominal hypertension to grade II, low abdominal perfusion. |
| AGI grade IV | Gastrointestinal failure with severe impact on other organ functions: acute gastrointestinal injuries progressing to a severe life-threatening condition with multiple organ dysfunction syndrome and exacerbation of shock | Clinical manifestations include intestinal ischemic necrosis, gastrointestinal bleeding leading to hemorrhagic shock, pseudocolonic obstruction, and abdominal septal compartment syndrome requiring decompression. |

**Table S3 Baseline comparisons of clinically relevant information between patients in derivation and validation cohorts (n=538)**

| Variable | Category | Derivation cohort  (n=387) | Validation cohort  (n=151) | *P* value |
| --- | --- | --- | --- | --- |
| Age (years), median (IQR) |  | 84 (73, 88) | 85 (76, 88) | 0.297 |
| Sex, n (%) |  |  |  | 0.096 |
|  | Female | 122 (31.5) | 59 (39.1) |  |
| Smoking, n (%) |  |  |  | 0.242 |
|  | Current | 56 (14.5) | 28 (18.5) |  |
| Drinking, n (%) |  |  |  | 0.381 |
|  | Current | 63 (16.3) | 20 (13.2) |  |
| Primary disease diagnosis, n (%) |  |  |  | 0.610 |
|  | Respiratory system | 216 (55.8) | 80 (53.0) |  |
|  | Circulatory system | 41 (10.6) | 14 (9.3) |  |
|  | Nervous system | 47 (12.1) | 20 (13.2) |  |
|  | Endocrine System | 2 (0.5) | 3 (2.0) |  |
|  | Urinary System | 10 (2.6) | 4 (2.6) |  |
|  | Systemic Reactions | 30 (7.8) | 9 (6.0) |  |
|  | Other | 41 (10.6) | 21 (13.9) |  |
| High blood pressure, n (%) |  |  |  | 0.457 |
|  | Yes | 243 (62.8) | 100 (66.2) |  |
| Diabetes, n (%) |  |  |  | 0.617 |
|  | Yes | 117 (30.2) | 49 (32.5) |  |
| Respiratory Failure, n (%) |  |  |  | 0.431 |
|  | Yes | 129 (33.3) | 45 (29.8) |  |
| Kidney Insufficiency, n (%) |  |  |  | 0.610 |
|  | Yes | 72 (18.6) | 31 (20.5) |  |
| Barthel index, n (%) |  |  |  | 0.589 |
|  | Severe/complete dependence | 286 (73.9) | 115 (76.2) |  |
| ACCI, median (IQR) |  | 6 (5, 7) | 7 (5, 8) | 0.092 |
| MEWS, median (IQR) |  | 2 (1, 3) | 2 (1, 3) | 0.138 |
| Oxygen partial pressure  (mmHg), n (%) |  |  |  | 0.532 |
|  | <80 | 247 (63.8) | 92 (60.9) |  |
| Serum sodium (mmol/L), n (%) |  |  |  | 0.310 |
|  | 135-145 | 216 (55.8) | 77 (51.0) |  |
|  | <135 | 156 (40.3) | 64 (42.4) |  |
|  | >145 | 15 (3.9) | 10 (6.6) |  |

**Table S3** (continued)

| Variable | Category | Derivation cohort  (n=387) | Validation cohort  (n=151) | *P* value |
| --- | --- | --- | --- | --- |
| Blood lactate (mmol/L), n (%) |  |  |  | 0.745 |
|  | >1.7 | 188 (48.6) | 71 (47.0) |  |
| Aspartate aminotransferase (IU/L), n (%) |  |  |  | 0.426 |
|  | >40 | 68 (17.6) | 31 (20.5) |  |
| Albumin (g/L), n (%) |  |  |  | 0.191 |
|  | <35 | 246 (63.6) | 105 (69.5) |  |
| Blood creatinine (μmol/L), n (%) |  |  |  | 0.325 |
|  | >133 | 56 (14.5) | 27 (17.9) |  |
| D-dimer (ng/ml), n (%) |  |  |  | 0.193 |
|  | >500 | 204 (52.7) | 89 (58.9) |  |
| White blood cells (×10^9^/L), n (%) |  |  |  | 0.263 |
|  | >10 | 97 (25.1) | 45 (29.8) |  |
| Hemoglobin (g/L), n (%) |  |  |  | 0.705 |
|  | <120 (male)/  <110 (female) | 247 (63.8) | 99 (65.6) |  |
| C-reactive protein (mg/L), n (%) |  |  |  | 0.877 |
|  | >10 | 287 (74.2) | 111 (73.5) |  |
| Mechanical ventilation, n (%) |  |  |  | 0.233 |
|  | Yes | 65 (16.8) | 32 (21.2) |  |
| Delayed enteral nutrition, n (%) |  |  |  | 0.894 |
|  | Yes | 187 (48.3) | 72 (47.7) |  |
| Number of vasoactive drugs(kinds), median (IQR) |  | 0 (0, 1) | 0 (0, 1) | 0.106 |
| Number of antibiotics (kinds), median (IQR) |  | 1 (0, 2) | 1 (1, 2) | 0.252 |
| Duration of antibiotic use (days), n (%) |  |  |  | 0.408 |
|  | >7 | 182 (47.0) | 77 (51.0) |  |
| Glucocorticoid, n (%) |  |  |  | 0.840 |
|  | Yes | 117 (30.2) | 47 (31.1) |  |
| Nonsteroidal anti-inflammatory medicine, n (%) |  |  |  | 0.274 |
|  | Yes | 95 (24.5) | 44 (29.1) |  |
| Sedative medication, n (%) |  |  |  | 0.089 |
|  | Yes | 136 (35.1) | 65 (43.0) |  |

*ACCI* age-corrected Charlson comorbidity index, *MEWS* modified early warning score

**Table S4 Multicollinearity diagnosis**

| Variable | Tolerance | Variance inflation factor |
| --- | --- | --- |
| Age | 0.764 | 1.308 |
| Diabetes | 0.850 | 1.177 |
| Barthel index | 0.713 | 1.403 |
| Modified early warning score | 0.686 | 1.459 |
| Age-corrected Charlson comorbidity index | 0.714 | 1.400 |
| Delayed enteral nutrition | 0.917 | 1.090 |
| Number of vasoactive drugs | 0.749 | 1.334 |
| Number of antibiotics | 0.522 | 1.914 |
| Duration of antibiotic use | 0.591 | 1.691 |
| Sedative medication | 0.894 | 1.118 |
| Serum sodium | 0.797 | 1.255 |
| Aspartate aminotransferase | 0.896 | 1.116 |
| Albumin | 0.723 | 1.383 |
| D-dimer | 0.774 | 1.292 |
| White blood cells | 0.846 | 1.183 |
| Hemoglobin | 0.755 | 1.324 |
| C-reactive protein | 0.776 | 1.289 |

**
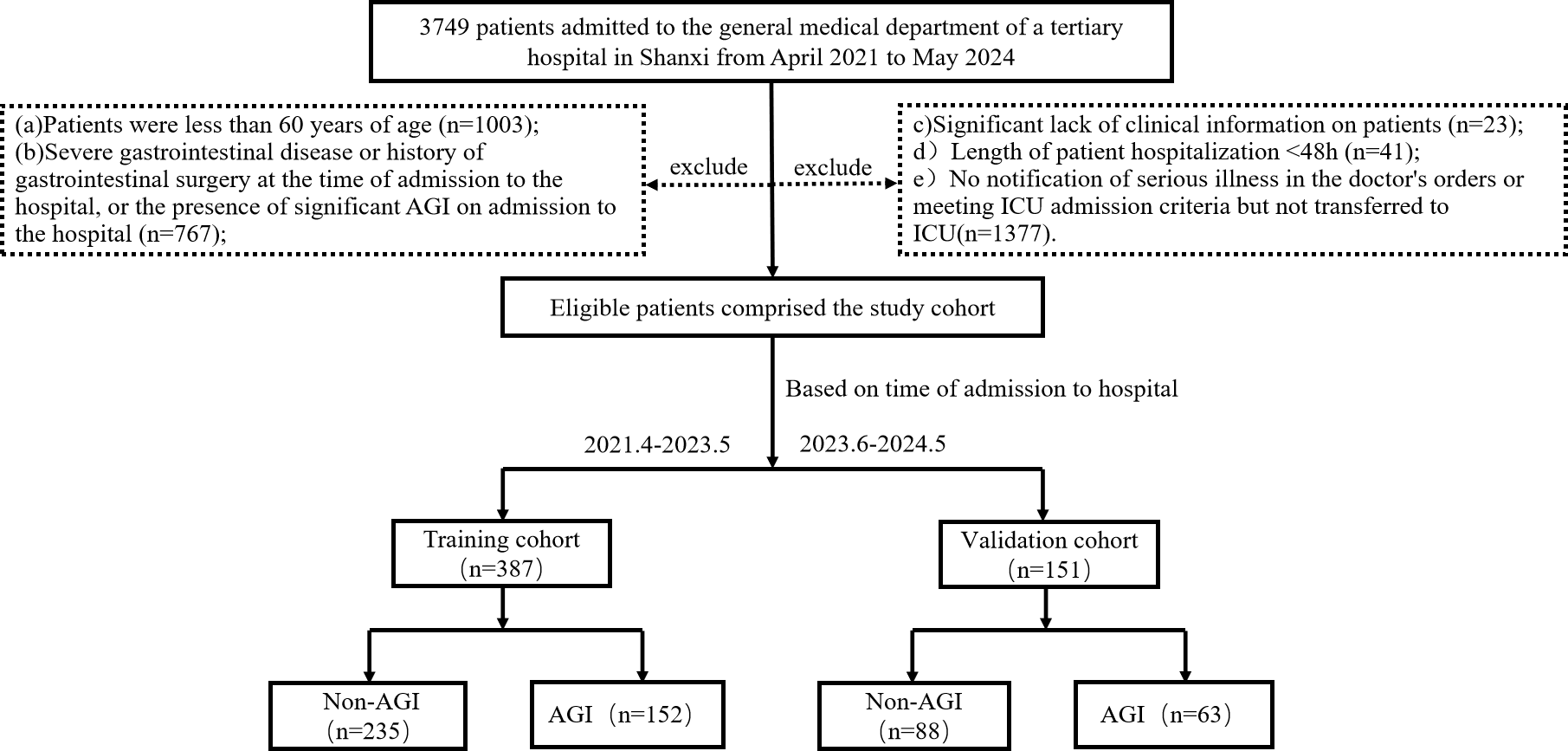
**

**Figure S1 Flowchart of patient recruitment**

**
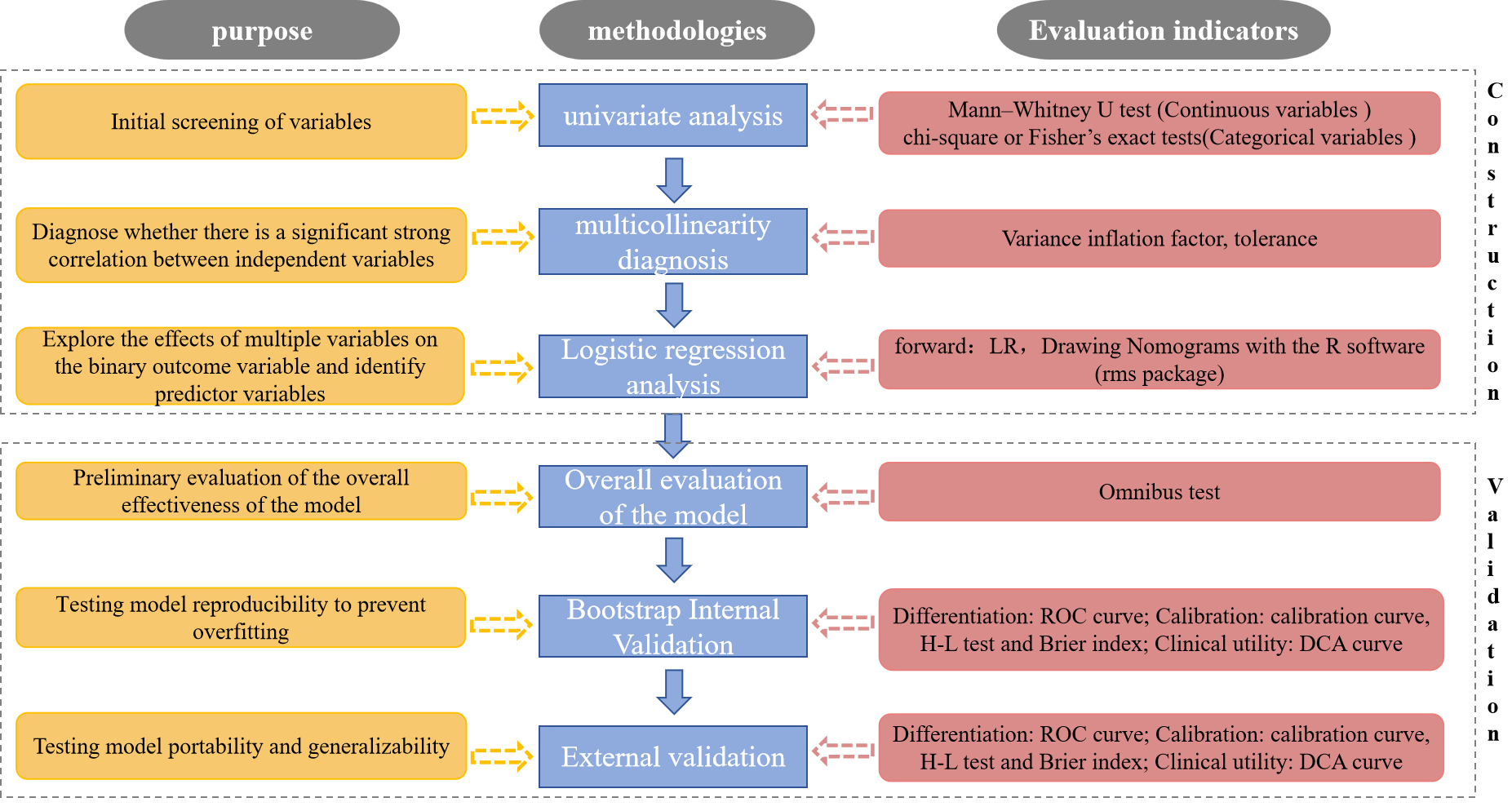
Figure S2 Flowchart of statistical methods**


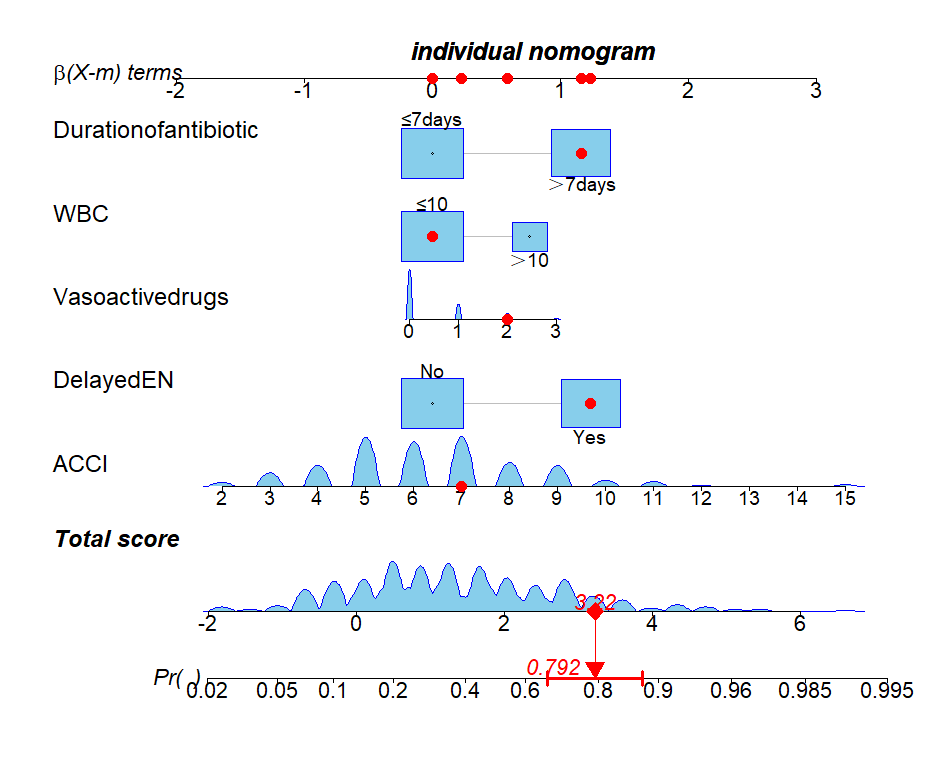
**Figure S3 Individual nomogram predicting the probability of AGI**

**
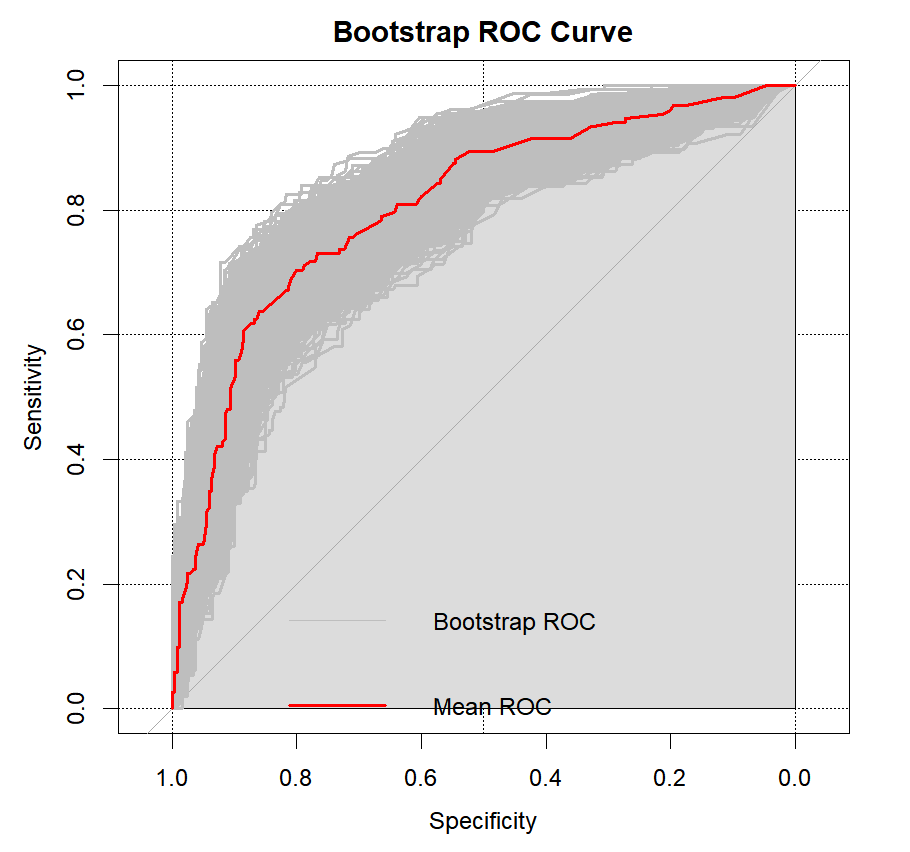
Figure S4 Receiver operating characteristic curve for the risk prediction model of AGI in the derivation cohort (Bootstrap 1000 times resampling method)**
